# Supplementary material for: Pyruvate Dehydrogenase Kinase 1 inhibition mediated oxidative phosphorylation enhancement in cartilage promotes osteoarthritis progression
Source: BMC Musculoskelet Disord. 2023 Jul 20;24:597. doi: 10.1186/s12891-023-06585-6 (PMC10357736; doi:10.1186/s12891-023-06585-6)
Supplement: Supplementary file 1 — Additional file 1. [file 12891_2023_6585_MOESM1_ESM.zip › MF.Gsea.1653627316242/GOMF_TRANSFERASE_ACTIVITY_TRANSFERRING_ONE_CARBON_GROUPS.html]

Details for gene set GOMF\_TRANSFERASE\_ACTIVITY\_TRANSFERRING\_ONE\_CARBON\_GROUPS[GSEA]

|  || Dataset | 55457\_collapsed\_to\_symbols.cls.cls #OA\_versus\_normal.cls.cls #OA\_versus\_normal\_repos |
| Phenotype | cls.cls#OA\_versus\_normal\_repos |
| Upregulated in class | OA |
| GeneSet | GOMF\_TRANSFERASE\_ACTIVITY\_TRANSFERRING\_ONE\_CARBON\_GROUPS |
| Enrichment Score (ES) | 0.23074414 |
| Normalized Enrichment Score (NES) | 1.151614 |
| Nominal p-value | 0.16564417 |
| FDR q-value | 0.6131303 |
| FWER p-Value | 1.0 |
Table: GSEA Results Summary

  

Fig 1: Enrichment plot: GOMF\_TRANSFERASE\_ACTIVITY\_TRANSFERRING\_ONE\_CARBON\_GROUPS      
 Profile of the Running ES Score & Positions of GeneSet Members on the Rank Ordered List

  

| SYMBOL | TITLE | RANK IN GENE LIST | RANK METRIC SCORE | RUNNING ES | CORE ENRICHMENT || 1 | TPMT | thiopurine S-methyltransferase [Source:HGNC Symbol;Acc:HGNC:12014] | 54 | 0.981 | 0.0157 | Yes |
| 2 | GATM | glycine amidinotransferase [Source:HGNC Symbol;Acc:HGNC:4175] | 56 | 0.976 | 0.0355 | Yes |
| 3 | TRMT5 | tRNA methyltransferase 5 [Source:HGNC Symbol;Acc:HGNC:23141] | 125 | 0.879 | 0.0480 | Yes |
| 4 | BUD23 | BUD23 rRNA methyltransferase and ribosome maturation factor [Source:HGNC Symbol;Acc:HGNC:16405] | 210 | 0.813 | 0.0579 | Yes |
| 5 | N6AMT1 | N-6 adenine-specific DNA methyltransferase 1 [Source:HGNC Symbol;Acc:HGNC:16021] | 330 | 0.734 | 0.0635 | Yes |
| 6 | COMT | catechol-O-methyltransferase [Source:HGNC Symbol;Acc:HGNC:2228] | 335 | 0.732 | 0.0780 | Yes |
| 7 | ATIC | 5-aminoimidazole-4-carboxamide ribonucleotide formyltransferase/IMP cyclohydrolase [Source:HGNC Symbol;Acc:HGNC:794] | 401 | 0.706 | 0.0873 | Yes |
| 8 | DNMT3A | DNA methyltransferase 3 alpha [Source:HGNC Symbol;Acc:HGNC:2978] | 406 | 0.704 | 0.1013 | Yes |
| 9 | ASH2L | "ASH2 like, histone lysine methyltransferase complex subunit [Source:HGNC Symbol;Acc:HGNC:744]" | 452 | 0.681 | 0.1116 | Yes |
| 10 | EED | embryonic ectoderm development [Source:HGNC Symbol;Acc:HGNC:3188] | 454 | 0.680 | 0.1254 | Yes |
| 11 | ANTKMT | adenine nucleotide translocase lysine methyltransferase [Source:HGNC Symbol;Acc:HGNC:14152] | 618 | 0.618 | 0.1251 | Yes |
| 12 | METTL18 | methyltransferase like 18 [Source:HGNC Symbol;Acc:HGNC:28793] | 685 | 0.597 | 0.1321 | Yes |
| 13 | HNMT | histamine N-methyltransferase [Source:HGNC Symbol;Acc:HGNC:5028] | 700 | 0.594 | 0.1430 | Yes |
| 14 | LCMT1 | leucine carboxyl methyltransferase 1 [Source:HGNC Symbol;Acc:HGNC:17557] | 735 | 0.585 | 0.1523 | Yes |
| 15 | HEMK1 | HemK methyltransferase family member 1 [Source:HGNC Symbol;Acc:HGNC:24923] | 742 | 0.583 | 0.1636 | Yes |
| 16 | DPH5 | diphthamide biosynthesis 5 [Source:HGNC Symbol;Acc:HGNC:24270] | 774 | 0.575 | 0.1729 | Yes |
| 17 | SMYD2 | SET and MYND domain containing 2 [Source:HGNC Symbol;Acc:HGNC:20982] | 856 | 0.553 | 0.1778 | Yes |
| 18 | LCMT2 | leucine carboxyl methyltransferase 2 [Source:HGNC Symbol;Acc:HGNC:17558] | 946 | 0.529 | 0.1815 | Yes |
| 19 | ARMT1 | acidic residue methyltransferase 1 [Source:HGNC Symbol;Acc:HGNC:17872] | 1080 | 0.500 | 0.1813 | Yes |
| 20 | ICMT | isoprenylcysteine carboxyl methyltransferase [Source:HGNC Symbol;Acc:HGNC:5350] | 1140 | 0.488 | 0.1866 | Yes |
| 21 | ASMTL | acetylserotonin O-methyltransferase like [Source:HGNC Symbol;Acc:HGNC:751] | 1143 | 0.487 | 0.1963 | Yes |
| 22 | PRMT2 | protein arginine methyltransferase 2 [Source:HGNC Symbol;Acc:HGNC:5186] | 1171 | 0.482 | 0.2040 | Yes |
| 23 | TRMT112 | tRNA methyltransferase activator subunit 11-2 [Source:HGNC Symbol;Acc:HGNC:26940] | 1359 | 0.449 | 0.1984 | Yes |
| 24 | GAMT | guanidinoacetate N-methyltransferase [Source:HGNC Symbol;Acc:HGNC:4136] | 1418 | 0.441 | 0.2028 | Yes |
| 25 | SHMT2 | serine hydroxymethyltransferase 2 [Source:HGNC Symbol;Acc:HGNC:10852] | 1518 | 0.426 | 0.2037 | Yes |
| 26 | RRP8 | ribosomal RNA processing 8 [Source:HGNC Symbol;Acc:HGNC:29030] | 1673 | 0.402 | 0.1998 | Yes |
| 27 | EMG1 | EMG1 N1-specific pseudouridine methyltransferase [Source:HGNC Symbol;Acc:HGNC:16912] | 1688 | 0.401 | 0.2069 | Yes |
| 28 | GCSH | glycine cleavage system protein H [Source:HGNC Symbol;Acc:HGNC:4208] | 1732 | 0.394 | 0.2115 | Yes |
| 29 | METTL9 | methyltransferase like 9 [Source:HGNC Symbol;Acc:HGNC:24586] | 1742 | 0.393 | 0.2188 | Yes |
| 30 | NSUN7 | NOP2/Sun RNA methyltransferase family member 7 [Source:HGNC Symbol;Acc:HGNC:25857] | 1830 | 0.381 | 0.2197 | Yes |
| 31 | MRM2 | mitochondrial rRNA methyltransferase 2 [Source:HGNC Symbol;Acc:HGNC:16352] | 1880 | 0.375 | 0.2235 | Yes |
| 32 | TRMT11 | tRNA methyltransferase 11 homolog [Source:HGNC Symbol;Acc:HGNC:21080] | 1885 | 0.374 | 0.2307 | Yes |
| 33 | METTL5 | "methyltransferase 5, N6-adenosine [Source:HGNC Symbol;Acc:HGNC:25006]" | 2107 | 0.349 | 0.2205 | No |
| 34 | COQ3 | "coenzyme Q3, methyltransferase [Source:HGNC Symbol;Acc:HGNC:18175]" | 2164 | 0.342 | 0.2230 | No |
| 35 | SMYD3 | SET and MYND domain containing 3 [Source:HGNC Symbol;Acc:HGNC:15513] | 2361 | 0.319 | 0.2142 | No |
| 36 | MGMT | O-6-methylguanine-DNA methyltransferase [Source:HGNC Symbol;Acc:HGNC:7059] | 2537 | 0.299 | 0.2065 | No |
| 37 | MEPCE | methylphosphate capping enzyme [Source:HGNC Symbol;Acc:HGNC:20247] | 2554 | 0.297 | 0.2113 | No |
| 38 | OTC | ornithine transcarbamylase [Source:HGNC Symbol;Acc:HGNC:8512] | 2628 | 0.287 | 0.2114 | No |
| 39 | FTSJ1 | FtsJ RNA 2'-O-methyltransferase 1 [Source:HGNC Symbol;Acc:HGNC:13254] | 2664 | 0.284 | 0.2144 | No |
| 40 | DNMT1 | DNA methyltransferase 1 [Source:HGNC Symbol;Acc:HGNC:2976] | 2836 | 0.267 | 0.2064 | No |
| 41 | FTCD | formimidoyltransferase cyclodeaminase [Source:HGNC Symbol;Acc:HGNC:3974] | 3064 | 0.244 | 0.1935 | No |
| 42 | TRMT12 | tRNA methyltransferase 12 homolog [Source:HGNC Symbol;Acc:HGNC:26091] | 3208 | 0.230 | 0.1870 | No |
| 43 | SETMAR | SET domain and mariner transposase fusion gene [Source:HGNC Symbol;Acc:HGNC:10762] | 3216 | 0.229 | 0.1911 | No |
| 44 | PRMT7 | protein arginine methyltransferase 7 [Source:HGNC Symbol;Acc:HGNC:25557] | 3250 | 0.226 | 0.1931 | No |
| 45 | METTL7A | methyltransferase like 7A [Source:HGNC Symbol;Acc:HGNC:24550] | 3409 | 0.210 | 0.1849 | No |
| 46 | EZH1 | enhancer of zeste 1 polycomb repressive complex 2 subunit [Source:HGNC Symbol;Acc:HGNC:3526] | 3565 | 0.197 | 0.1768 | No |
| 47 | NSUN3 | NOP2/Sun RNA methyltransferase 3 [Source:HGNC Symbol;Acc:HGNC:26208] | 3612 | 0.192 | 0.1771 | No |
| 48 | PCMT1 | protein-L-isoaspartate (D-aspartate) O-methyltransferase [Source:HGNC Symbol;Acc:HGNC:8728] | 3925 | 0.162 | 0.1559 | No |
| 49 | PCMTD2 | protein-L-isoaspartate (D-aspartate) O-methyltransferase domain containing 2 [Source:HGNC Symbol;Acc:HGNC:15882] | 3975 | 0.158 | 0.1553 | No |
| 50 | TRMT1L | tRNA methyltransferase 1 like [Source:HGNC Symbol;Acc:HGNC:16782] | 4007 | 0.155 | 0.1560 | No |
| 51 | CIAPIN1 | cytokine induced apoptosis inhibitor 1 [Source:HGNC Symbol;Acc:HGNC:28050] | 4161 | 0.141 | 0.1468 | No |
| 52 | FBL | fibrillarin [Source:HGNC Symbol;Acc:HGNC:3599] | 4219 | 0.135 | 0.1451 | No |
| 53 | TYMS | thymidylate synthetase [Source:HGNC Symbol;Acc:HGNC:12441] | 4328 | 0.126 | 0.1392 | No |
| 54 | PEMT | phosphatidylethanolamine N-methyltransferase [Source:HGNC Symbol;Acc:HGNC:8830] | 4496 | 0.113 | 0.1284 | No |
| 55 | SETD3 | "SET domain containing 3, actin histidine methyltransferase [Source:HGNC Symbol;Acc:HGNC:20493]" | 4506 | 0.113 | 0.1300 | No |
| 56 | SMYD5 | SMYD family member 5 [Source:HGNC Symbol;Acc:HGNC:16258] | 4769 | 0.089 | 0.1112 | No |
| 57 | MRM3 | mitochondrial rRNA methyltransferase 3 [Source:HGNC Symbol;Acc:HGNC:18485] | 4806 | 0.087 | 0.1102 | No |
| 58 | SETDB1 | SET domain bifurcated histone lysine methyltransferase 1 [Source:HGNC Symbol;Acc:HGNC:10761] | 4891 | 0.080 | 0.1052 | No |
| 59 | FAM86C1P | "family with sequence similarity 86 member C1, pseudogene [Source:HGNC Symbol;Acc:HGNC:25561]" | 4906 | 0.079 | 0.1057 | No |
| 60 | ZCCHC4 | zinc finger CCHC-type containing 4 [Source:HGNC Symbol;Acc:HGNC:22917] | 4909 | 0.079 | 0.1072 | No |
| 61 | FAM98A | family with sequence similarity 98 member A [Source:HGNC Symbol;Acc:HGNC:24520] | 5093 | 0.065 | 0.0941 | No |
| 62 | NSD1 | nuclear receptor binding SET domain protein 1 [Source:HGNC Symbol;Acc:HGNC:14234] | 5178 | 0.058 | 0.0887 | No |
| 63 | PCIF1 | phosphorylated CTD interacting factor 1 [Source:HGNC Symbol;Acc:HGNC:16200] | 5319 | 0.046 | 0.0787 | No |
| 64 | NSUN5 | NOP2/Sun RNA methyltransferase 5 [Source:HGNC Symbol;Acc:HGNC:16385] | 5434 | 0.038 | 0.0705 | No |
| 65 | EHMT2 | euchromatic histone lysine methyltransferase 2 [Source:HGNC Symbol;Acc:HGNC:14129] | 5507 | 0.032 | 0.0655 | No |
| 66 | MECOM | MDS1 and EVI1 complex locus [Source:HGNC Symbol;Acc:HGNC:3498] | 5622 | 0.022 | 0.0570 | No |
| 67 | METTL17 | methyltransferase like 17 [Source:HGNC Symbol;Acc:HGNC:19280] | 5673 | 0.018 | 0.0534 | No |
| 68 | TRMT9B | tRNA methyltransferase 9B (putative) [Source:HGNC Symbol;Acc:HGNC:26725] | 5702 | 0.016 | 0.0515 | No |
| 69 | METTL8 | "methyltransferase 8, methylcytidine [Source:HGNC Symbol;Acc:HGNC:25856]" | 5706 | 0.015 | 0.0516 | No |
| 70 | TFB1M | "transcription factor B1, mitochondrial [Source:HGNC Symbol;Acc:HGNC:17037]" | 5789 | 0.009 | 0.0454 | No |
| 71 | PRDM11 | PR/SET domain 11 [Source:HGNC Symbol;Acc:HGNC:13996] | 5876 | 0.004 | 0.0387 | No |
| 72 | THUMPD2 | THUMP domain containing 2 [Source:HGNC Symbol;Acc:HGNC:14890] | 5913 | 0.001 | 0.0359 | No |
| 73 | METTL22 | "methyltransferase 22, Kin17 lysine [Source:HGNC Symbol;Acc:HGNC:28368]" | 5921 | 0.001 | 0.0354 | No |
| 74 | PRDM10 | PR/SET domain 10 [Source:HGNC Symbol;Acc:HGNC:13995] | 5932 | 0.000 | 0.0346 | No |
| 75 | ASMT | acetylserotonin O-methyltransferase [Source:HGNC Symbol;Acc:HGNC:750] | 5986 | -0.003 | 0.0305 | No |
| 76 | SUV39H1 | suppressor of variegation 3-9 homolog 1 [Source:HGNC Symbol;Acc:HGNC:11479] | 6028 | -0.007 | 0.0274 | No |
| 77 | BHMT2 | betaine--homocysteine S-methyltransferase 2 [Source:HGNC Symbol;Acc:HGNC:1048] | 6165 | -0.018 | 0.0171 | No |
| 78 | NDUFAF5 | NADH:ubiquinone oxidoreductase complex assembly factor 5 [Source:HGNC Symbol;Acc:HGNC:15899] | 6167 | -0.018 | 0.0174 | No |
| 79 | KMT5B | lysine methyltransferase 5B [Source:HGNC Symbol;Acc:HGNC:24283] | 6198 | -0.020 | 0.0154 | No |
| 80 | TRDMT1 | tRNA aspartic acid methyltransferase 1 [Source:HGNC Symbol;Acc:HGNC:2977] | 6204 | -0.021 | 0.0155 | No |
| 81 | NSD3 | nuclear receptor binding SET domain protein 3 [Source:HGNC Symbol;Acc:HGNC:12767] | 6220 | -0.022 | 0.0147 | No |
| 82 | TRMT61B | tRNA methyltransferase 61B [Source:HGNC Symbol;Acc:HGNC:26070] | 6223 | -0.022 | 0.0150 | No |
| 83 | PRMT1 | protein arginine methyltransferase 1 [Source:HGNC Symbol;Acc:HGNC:5187] | 6319 | -0.029 | 0.0082 | No |
| 84 | CAD | "carbamoyl-phosphate synthetase 2, aspartate transcarbamylase, and dihydroorotase [Source:HGNC Symbol;Acc:HGNC:1424]" | 6359 | -0.032 | 0.0058 | No |
| 85 | METTL2B | "methyltransferase 2B, methylcytidine [Source:HGNC Symbol;Acc:HGNC:18272]" | 6522 | -0.044 | -0.0061 | No |
| 86 | EZH2 | enhancer of zeste 2 polycomb repressive complex 2 subunit [Source:HGNC Symbol;Acc:HGNC:3527] | 6562 | -0.047 | -0.0082 | No |
| 87 | METTL3 | "methyltransferase 3, N6-adenosine-methyltransferase complex catalytic subunit [Source:HGNC Symbol;Acc:HGNC:17563]" | 6685 | -0.055 | -0.0166 | No |
| 88 | METTL25B | methyltransferase like 25B [Source:HGNC Symbol;Acc:HGNC:24273] | 6774 | -0.061 | -0.0223 | No |
| 89 | TRMT2B | tRNA methyltransferase 2 homolog B [Source:HGNC Symbol;Acc:HGNC:25748] | 6855 | -0.068 | -0.0272 | No |
| 90 | SETD2 | "SET domain containing 2, histone lysine methyltransferase [Source:HGNC Symbol;Acc:HGNC:18420]" | 6926 | -0.074 | -0.0312 | No |
| 91 | TFB2M | "transcription factor B2, mitochondrial [Source:HGNC Symbol;Acc:HGNC:18559]" | 7112 | -0.089 | -0.0439 | No |
| 92 | EEF2KMT | eukaryotic elongation factor 2 lysine methyltransferase [Source:HGNC Symbol;Acc:HGNC:32221] | 7177 | -0.094 | -0.0470 | No |
| 93 | BHMT | betaine--homocysteine S-methyltransferase [Source:HGNC Symbol;Acc:HGNC:1047] | 7250 | -0.099 | -0.0506 | No |
| 94 | NSD2 | nuclear receptor binding SET domain protein 2 [Source:HGNC Symbol;Acc:HGNC:12766] | 7622 | -0.129 | -0.0771 | No |
| 95 | TGS1 | trimethylguanosine synthase 1 [Source:HGNC Symbol;Acc:HGNC:17843] | 7791 | -0.141 | -0.0875 | No |
| 96 | PRMT5 | protein arginine methyltransferase 5 [Source:HGNC Symbol;Acc:HGNC:10894] | 7904 | -0.148 | -0.0932 | No |
| 97 | CMTR1 | cap methyltransferase 1 [Source:HGNC Symbol;Acc:HGNC:21077] | 7951 | -0.151 | -0.0938 | No |
| 98 | TARBP1 | TAR (HIV-1) RNA binding protein 1 [Source:HGNC Symbol;Acc:HGNC:11568] | 8091 | -0.163 | -0.1014 | No |
| 99 | CARM1 | coactivator associated arginine methyltransferase 1 [Source:HGNC Symbol;Acc:HGNC:23393] | 8131 | -0.165 | -0.1011 | No |
| 100 | PRMT8 | protein arginine methyltransferase 8 [Source:HGNC Symbol;Acc:HGNC:5188] | 8150 | -0.167 | -0.0991 | No |
| 101 | CAMKMT | calmodulin-lysine N-methyltransferase [Source:HGNC Symbol;Acc:HGNC:26276] | 8484 | -0.193 | -0.1213 | No |
| 102 | PRDM5 | PR/SET domain 5 [Source:HGNC Symbol;Acc:HGNC:9349] | 8486 | -0.193 | -0.1175 | No |
| 103 | SUV39H2 | suppressor of variegation 3-9 homolog 2 [Source:HGNC Symbol;Acc:HGNC:17287] | 8529 | -0.196 | -0.1168 | No |
| 104 | TRMO | tRNA methyltransferase O [Source:HGNC Symbol;Acc:HGNC:30967] | 8541 | -0.197 | -0.1137 | No |
| 105 | PRDM14 | PR/SET domain 14 [Source:HGNC Symbol;Acc:HGNC:14001] | 8553 | -0.198 | -0.1105 | No |
| 106 | KMT2A | lysine methyltransferase 2A [Source:HGNC Symbol;Acc:HGNC:7132] | 8807 | -0.217 | -0.1260 | No |
| 107 | DOT1L | DOT1 like histone lysine methyltransferase [Source:HGNC Symbol;Acc:HGNC:24948] | 8832 | -0.220 | -0.1234 | No |
| 108 | NSUN5P1 | NSUN5 pseudogene 1 [Source:HGNC Symbol;Acc:HGNC:19146] | 8988 | -0.231 | -0.1308 | No |
| 109 | PRDM2 | PR/SET domain 2 [Source:HGNC Symbol;Acc:HGNC:9347] | 8992 | -0.231 | -0.1264 | No |
| 110 | AMT | aminomethyltransferase [Source:HGNC Symbol;Acc:HGNC:473] | 9021 | -0.234 | -0.1238 | No |
| 111 | PRMT3 | protein arginine methyltransferase 3 [Source:HGNC Symbol;Acc:HGNC:30163] | 9034 | -0.234 | -0.1200 | No |
| 112 | MRM1 | mitochondrial rRNA methyltransferase 1 [Source:HGNC Symbol;Acc:HGNC:26202] | 9114 | -0.241 | -0.1213 | No |
| 113 | FTSJ3 | FtsJ RNA 2'-O-methyltransferase 3 [Source:HGNC Symbol;Acc:HGNC:17136] | 9193 | -0.247 | -0.1224 | No |
| 114 | GNMT | glycine N-methyltransferase [Source:HGNC Symbol;Acc:HGNC:4415] | 9208 | -0.248 | -0.1185 | No |
| 115 | MTR | 5-methyltetrahydrofolate-homocysteine methyltransferase [Source:HGNC Symbol;Acc:HGNC:7468] | 9213 | -0.249 | -0.1137 | No |
| 116 | ALDH1L1 | aldehyde dehydrogenase 1 family member L1 [Source:HGNC Symbol;Acc:HGNC:3978] | 9302 | -0.256 | -0.1154 | No |
| 117 | SETD4 | SET domain containing 4 [Source:HGNC Symbol;Acc:HGNC:1258] | 9452 | -0.269 | -0.1217 | No |
| 118 | PRDM13 | PR/SET domain 13 [Source:HGNC Symbol;Acc:HGNC:13998] | 9484 | -0.273 | -0.1186 | No |
| 119 | WDR4 | WD repeat domain 4 [Source:HGNC Symbol;Acc:HGNC:12756] | 9631 | -0.286 | -0.1242 | No |
| 120 | SHMT1 | serine hydroxymethyltransferase 1 [Source:HGNC Symbol;Acc:HGNC:10850] | 9727 | -0.294 | -0.1257 | No |
| 121 | GART | "phosphoribosylglycinamide formyltransferase, phosphoribosylglycinamide synthetase, phosphoribosylaminoimidazole synthetase [Source:HGNC Symbol;Acc:HGNC:4163]" | 9861 | -0.307 | -0.1299 | No |
| 122 | PNMT | phenylethanolamine N-methyltransferase [Source:HGNC Symbol;Acc:HGNC:9160] | 9991 | -0.320 | -0.1335 | No |
| 123 | DIMT1 | DIM1 rRNA methyltransferase and ribosome maturation factor [Source:HGNC Symbol;Acc:HGNC:30217] | 10023 | -0.324 | -0.1293 | No |
| 124 | METTL13 | "methyltransferase 13, eEF1A lysine and N-terminal methyltransferase [Source:HGNC Symbol;Acc:HGNC:24248]" | 10053 | -0.326 | -0.1250 | No |
| 125 | NDUFAF7 | NADH:ubiquinone oxidoreductase complex assembly factor 7 [Source:HGNC Symbol;Acc:HGNC:28816] | 10077 | -0.328 | -0.1201 | No |
| 126 | DNMT3B | DNA methyltransferase 3 beta [Source:HGNC Symbol;Acc:HGNC:2979] | 10089 | -0.330 | -0.1143 | No |
| 127 | SUZ12 | SUZ12 polycomb repressive complex 2 subunit [Source:HGNC Symbol;Acc:HGNC:17101] | 10104 | -0.331 | -0.1086 | No |
| 128 | PRDM16 | PR/SET domain 16 [Source:HGNC Symbol;Acc:HGNC:14000] | 10211 | -0.342 | -0.1100 | No |
| 129 | NNMT | nicotinamide N-methyltransferase [Source:HGNC Symbol;Acc:HGNC:7861] | 10216 | -0.342 | -0.1034 | No |
| 130 | METTL1 | "methyltransferase 1, tRNA methylguanosine [Source:HGNC Symbol;Acc:HGNC:7030]" | 10333 | -0.354 | -0.1053 | No |
| 131 | PRDM1 | PR/SET domain 1 [Source:HGNC Symbol;Acc:HGNC:9346] | 10374 | -0.358 | -0.1011 | No |
| 132 | TRMT61A | tRNA methyltransferase 61A [Source:HGNC Symbol;Acc:HGNC:23790] | 10603 | -0.384 | -0.1112 | No |
| 133 | PRDM9 | PR/SET domain 9 [Source:HGNC Symbol;Acc:HGNC:13994] | 10798 | -0.405 | -0.1182 | No |
| 134 | CXXC1 | CXXC finger protein 1 [Source:HGNC Symbol;Acc:HGNC:24343] | 10853 | -0.411 | -0.1141 | No |
| 135 | WDR82 | WD repeat domain 82 [Source:HGNC Symbol;Acc:HGNC:28826] | 10854 | -0.411 | -0.1058 | No |
| 136 | TTLL12 | tubulin tyrosine ligase like 12 [Source:HGNC Symbol;Acc:HGNC:28974] | 10861 | -0.411 | -0.0979 | No |
| 137 | VCPKMT | valosin containing protein lysine methyltransferase [Source:HGNC Symbol;Acc:HGNC:20352] | 10888 | -0.416 | -0.0915 | No |
| 138 | TRMT2A | tRNA methyltransferase 2 homolog A [Source:HGNC Symbol;Acc:HGNC:24974] | 10950 | -0.424 | -0.0876 | No |
| 139 | PRDM4 | PR/SET domain 4 [Source:HGNC Symbol;Acc:HGNC:9348] | 11098 | -0.443 | -0.0902 | No |
| 140 | SETD1B | "SET domain containing 1B, histone lysine methyltransferase [Source:HGNC Symbol;Acc:HGNC:29187]" | 11235 | -0.461 | -0.0915 | No |
| 141 | METTL16 | "methyltransferase 16, N6-methyladenosine [Source:HGNC Symbol;Acc:HGNC:28484]" | 11346 | -0.478 | -0.0904 | No |
| 142 | RNMT | RNA guanine-7 methyltransferase [Source:HGNC Symbol;Acc:HGNC:10075] | 11392 | -0.485 | -0.0840 | No |
| 143 | SETD6 | "SET domain containing 6, protein lysine methyltransferase [Source:HGNC Symbol;Acc:HGNC:26116]" | 11467 | -0.498 | -0.0797 | No |
| 144 | KMT2D | lysine methyltransferase 2D [Source:HGNC Symbol;Acc:HGNC:7133] | 11590 | -0.514 | -0.0789 | No |
| 145 | FBXO11 | F-box protein 11 [Source:HGNC Symbol;Acc:HGNC:13590] | 11598 | -0.516 | -0.0689 | No |
| 146 | PRDM12 | PR/SET domain 12 [Source:HGNC Symbol;Acc:HGNC:13997] | 11673 | -0.528 | -0.0640 | No |
| 147 | EHMT1 | euchromatic histone lysine methyltransferase 1 [Source:HGNC Symbol;Acc:HGNC:24650] | 11686 | -0.530 | -0.0542 | No |
| 148 | TRMT1 | tRNA methyltransferase 1 [Source:HGNC Symbol;Acc:HGNC:25980] | 11775 | -0.545 | -0.0500 | No |
| 149 | TRMT13 | tRNA methyltransferase 13 homolog [Source:HGNC Symbol;Acc:HGNC:25502] | 11792 | -0.548 | -0.0402 | No |
| 150 | RBBP5 | "RB binding protein 5, histone lysine methyltransferase complex subunit [Source:HGNC Symbol;Acc:HGNC:9888]" | 11816 | -0.553 | -0.0307 | No |
| 151 | METTL4 | "methyltransferase 4, N6-adenosine [Source:HGNC Symbol;Acc:HGNC:24726]" | 11976 | -0.589 | -0.0312 | No |
| 152 | PRDM8 | PR/SET domain 8 [Source:HGNC Symbol;Acc:HGNC:13993] | 12112 | -0.623 | -0.0292 | No |
| 153 | ECE2 | endothelin converting enzyme 2 [Source:HGNC Symbol;Acc:HGNC:13275] | 12319 | -0.682 | -0.0315 | No |
| 154 | SETD5 | SET domain containing 5 [Source:HGNC Symbol;Acc:HGNC:25566] | 12339 | -0.688 | -0.0190 | No |
| 155 | NOP2 | NOP2 nucleolar protein [Source:HGNC Symbol;Acc:HGNC:7867] | 12372 | -0.699 | -0.0073 | No |
| 156 | ASH1L | ASH1 like histone lysine methyltransferase [Source:HGNC Symbol;Acc:HGNC:19088] | 12405 | -0.708 | 0.0046 | No |
| 157 | KMT2B | lysine methyltransferase 2B [Source:HGNC Symbol;Acc:HGNC:15840] | 12446 | -0.723 | 0.0162 | No |
| 158 | SETD1A | "SET domain containing 1A, histone lysine methyltransferase [Source:HGNC Symbol;Acc:HGNC:29010]" | 12793 | -0.954 | 0.0084 | No |
Table: GSEA details [plain text format]

  

Fig 2: GOMF\_TRANSFERASE\_ACTIVITY\_TRANSFERRING\_ONE\_CARBON\_GROUPS      
 Blue-Pink O' Gram in the Space of the Analyzed GeneSet

  

Fig 3: GOMF\_TRANSFERASE\_ACTIVITY\_TRANSFERRING\_ONE\_CARBON\_GROUPS: Random ES distribution      
 Gene set null distribution of ES for **GOMF\_TRANSFERASE\_ACTIVITY\_TRANSFERRING\_ONE\_CARBON\_GROUPS**
